# Effects of THC, CBD, and Their Combination on EEG Dynamics in Rats

**DOI:** 10.33549/physiolres.935771

**Published:** 2026-04-01

**Authors:** Marcel BOCHIN, Čestmír VEJMOLA, Vlastimil KOUDELKA, Stanislav JIŘÍČEK, Marek NIKOLIČ, Klára ŠÍCHOVÁ, Tomáš PÁLENÍČEK

**Affiliations:** 1 National Institute of Mental Health, Klecany, Czech Republic; 2 Faculty of Science, Charles University, Prague, Czech Republic; 3 Department of Cybernetics, Faculty of Electrical Engineering, Czech Technical University in Prague, Prague, Czech Republic; 4 Department of Complex Systems, Institute of Computer Science of the Czech Academy of Sciences, Prague, Czech Republic; 5 Third Faculty of Medicine, Charles University, Prague, Czech Republic

**Keywords:** Cannabinoids, Electroencephalography, Rats, Spectral Analysis, Functional Connectivity

## Abstract

Cannabinoids modulate brain network activity, yet the spatial organization and temporal evolution of their electrophysiological effects remain insufficiently characterized in animal models. Here, we investigated how acute oral administration of Δ9-tetrahydrocannabinol (THC; 10 mg/kg), cannabidiol (CBD; 10 mg/kg), and their combination influences resting-state EEG dynamics in freely moving rats. Adult male Wistar rats implanted with a 12-electrode epidural array received one of the treatments via intragastric gavage. EEG was recorded during baseline and three post-administration intervals (80–90, 110–120, 140–150 min) and analyzed using spectral decomposition, source localization, and functional connectivity within an anatomically realistic rat head model. Cannabinoid administration increased low-frequency spectral power, with source-space statistics identifying significant delta–beta clusters (p < 0.05, FDR-corrected), most prominently in prefrontal, cingulate, hippocampal, and striatal regions. THC and THC+CBD produced the strongest and most spatially extensive significant effects, whereas CBD yielded fewer and smaller clusters. Connectivity analyses demonstrated convergent treatment-related changes, revealing significant alterations in functional coupling within overlapping regions rich in CB1 receptors. These connectivity effects mirrored the spatial distribution of source-power findings, indicating that cannabinoids influence not only local oscillatory activity but also large-scale network organization. Across analyses, the combination treatment closely resembled the THC profile, suggesting a dominant THC-driven contribution with CBD exerting weaker, directionally similar modulation. Together, these findings identify a robust cannabinoid-specific EEG signature characterized by enhanced low-frequency oscillatory activity, altered functional connectivity, and regionally selective engagement of CB1-rich cortical and limbic structures.

## Introduction

Δ9-tetrahydrocannabinol (THC) and cannabidiol (CBD) represent the two principal phytocannabinoids derived from *Cannabis sativa*, each exerting distinct and often opposing effects on brain function, cognition, and behavior [1,2]. THC, the primary psychoactive component, acts as a partial agonist at cannabinoid type 1 (CB1) receptors, which are abundantly expressed in cortical and limbic regions [3]. Through CB1 receptor activation, THC modulates GABAergic and glutamatergic neurotransmission, leading to alterations in synaptic plasticity and neural oscillatory activity [4]. In contrast, CBD exhibits a complex pharmacological profile, with low affinity for CB1 and CB2 receptors. Instead, it acts through multiple mechanisms, including modulation of serotonergic and vanilloid receptors, inhibition of anandamide reuptake and fatty acid amide hydrolase (FAAH) [5,6], and partial agonism at dopamine D3 receptors [7]. These distinct receptor mechanisms translate into divergent neurophysiological and behavioral outcomes, with THC typically associated with psychotomimetic effects and cognitive impairment, while CBD demonstrates anxiolytic, anticonvulsant, and neuroprotective properties [8,9].

Quantitative electroencephalography (qEEG) is a powerful tool for characterizing the neurophysiological effects of psychoactive substances. In humans, resting-state EEG studies have revealed that chronic cannabis users exhibit elevated alpha power (8–12 Hz) compared to controls, with these increases most prominent over prefrontal cortical regions [10]. Acute cannabis consumption produces increased alpha and beta activity in prefrontal, frontal, and temporal regions [11]. A recent high-density EEG study examining the administration of combined THC and CBD in patients with insomnia reported regional increases in alpha and beta activity during REM sleep, alongside alterations in other frequency bands [12].

Beyond resting-state alterations, acute THC administration disrupts task-related neural oscillations in humans. Intravenous THC (1.25 mg) produces significant reductions in theta power (3.5–7 Hz) during working memory tasks, with these changes correlating with cognitive performance impairments [13]. THC also induces dose-dependent reductions in frontal theta coherence, and the magnitude of this coherence disruption strongly correlates with the emergence of positive psychotic symptoms [14]. Perhaps most robustly, THC disrupts gamma-band oscillations, with intravenous administration reducing 40 Hz auditory steady-state response (ASSR) intertrial coherence and evoked power, effects that correlate with psychosis-relevant symptomatology [15]. These findings suggest that THC alters oscillatory dynamics by increasing spectral power in lower frequency bands, particularly in prefrontal regions.

Parallel investigations in rodent models have corroborated and extended these human findings. Acute vaporized THC in rats produces pronounced suppression of gamma spectral power (>32–100 Hz) across prefrontal cortex, orbitofrontal cortex, and dorsal striatum, with many of these changes persisting for at least seven days following a single exposure [16]. High-dose oral THC (20 mg/kg) produces time-dependent suppression of gamma power in cingulate cortex, dorsal hippocampus, and nucleus accumbens, with effects emerging approximately two hours post-administration and persisting up to 24 hours [17]. Beyond spectral power, THC reduces gamma coherence between orbitofrontal-prefrontal and dorsal striatum-prefrontal electrode pairs, indicating disruption of long-range synchronization across cortico-striatal circuits [16]. Synthetic cannabinoid receptor agonists produce prominent increases in the 5.0–6.0 Hz (theta) range in rats, suggesting that cannabinoids can produce bidirectional effects on oscillatory power depending on frequency band and recording conditions [18].

The interaction between THC and CBD represents a critical area of investigation, with emerging evidence suggesting that CBD can modulate or attenuate certain THC-induced neurophysiological changes. In human fMRI studies, while THC alone (8 mg inhaled) disrupts striato-cortical connectivity in associative, sensorimotor, and limbic subdivisions, co-administration with CBD (10 mg) mitigates THC’s effects specifically in the limbic striatal network, restoring connectivity to placebo levels [19]. However, the nature of THC-CBD interactions appears to be complex and dose-ratio-dependent. In the sleep study mentioned earlier, combined oral administration of CBD and THC (200 mg CBD + 10 mg THC) reduced REM sleep time by 33.9 minutes and altered regional spectral power, with decreases in gamma during N2 sleep, decreases in delta during N3 sleep, and increases in parietal alpha during REM [12]. These findings indicate that the combination produces unique effects on sleep architecture and regional oscillatory activity that differ from either compound alone. Moreover, while CBD has demonstrated clear effects in clinical populations and disease models, direct evidence for CBD-induced alterations in resting-state spectral power in healthy populations remains relatively limited. Neuroimaging studies suggest that CBD’s neurophysiological effects may be more subtle or state-dependent than those of THC [20,21].

Despite the growing body of human and animal cannabinoid research, significant methodological gaps remain. Most rodent studies employ limited electrode numbers, which preclude the precise anatomical localization of cannabinoid effects. Source localization approaches, which utilize scalp-recorded electrical activity to estimate the anatomical origins of neural signals, have been successfully applied in human cannabinoid research [10,20]. However, their application in animal models remains limited. This represents a critical translational gap, as source-level analysis provides an informative approach for understanding how cannabinoids alter activity in specific brain structures and networks. By applying source localization methods to multi-electrode recordings in rats, it becomes possible to directly compare regional cannabinoid effects across species and to identify conserved neural substrates of cannabinoid action.

The present study addresses this methodological gap by investigating the independent and combined effects of THC (10 mg/kg), CBD (10 mg/kg), and their combination on EEG dynamics in rats using a comprehensive analytical framework. Recordings were obtained from 12 electrodes distributed across the rat brain, providing spatial coverage that enables application of source localization algorithms to identify deep anatomical generators of oscillatory activity. We examined spectral power across delta, theta, alpha, beta, and gamma frequency bands, localized power changes to specific brain regions, and calculated functional connectivity networks. This translational approach enables a direct comparison of our findings with the growing body of human qEEG literature.

## Methods

### Animals

Experiments were conducted on adult, experimentally naïve male Wistar rats (Velaz s.r.o., Konárovice, Czech Republic). Each animal was used for a single recording session. Upon arrival, rats were acclimatized to the animal facility for at least one week before surgery. Animals were housed in pairs in standard polycarbonate cages under a 12-h light/dark cycle (lights on at 06:00), at a controlled temperature of 21–24 °C and relative humidity of approximately 60 %. Food (standard ST2 pellets) and water were available ad libitum. All experimental procedures were approved by the Expert Committee for the Protection of Experimental Animals of the 3rd Faculty of Medicine, Charles University (Prague, Czech Republic) and were conducted in accordance with the Czech Animal Protection Act and the EU Council Directive 86/609/EU.

### Stereotactic surgery

Rats were anesthetized with isoflurane (2.5–3 %) and positioned in a stereotactic frame (Stoelting) using atraumatic ear bars. The scalp was shaved, disinfected with Betadine, incised, and the periosteum removed. Electrode placement coordinates were referenced to bregma and determined from the Paxinos and Watson Rat Brain Atlas [22]: frontal association cortex (F3/F4: A + 5.0 mm, L ± 2.0 mm), primary motor cortex (C3/C4: A + 2.2 mm, L ± 3.2 mm), medial parietal association cortex (P3/P4: A − 3.8 mm, L ± 2.5 mm), lateral parietal association cortex (P5/P6: A − 4.5 mm, L ± 4.5 mm), secondary auditory cortex (T3/T4: A − 3.6 mm, L ± 7.2 mm), and temporal association cortex (T5/T6: A − 8.3 mm, L ± 5.8 mm). Cranial openings (0.5 mm) were drilled with a Meisinger micro-drill, and gold-plated epidural electrodes (Mill-Max; 0.48 mm shaft) were implanted. The reference electrode was positioned above the olfactory bulb, and a ground electrode was placed subcutaneously in the occipital region. All electrodes were secured using Dentalon®, cold-cure acrylic cement. Animals recovered uneventfully, with no signs of infection or weight loss, and were housed singly thereafter to protect the implant. Twenty-four hours prior to the recording session, the head-stage connector was attached under brief isoflurane anesthesia.

### Data collection

Recordings were performed seven days after surgery, during the light phase of the circadian cycle. Animals were connected to the EEG system in their home cages approximately 10 min before data acquisition to minimize handling-related stress. Each session commenced with a 10-min drug-free baseline period, followed by pharmacological administration. THC and CBD were dissolved in sunflower oil and administered intragastrically at a dose of 10 mg/kg. This dose was chosen within the range commonly used in rodent studies investigating behavioural and neurophysiological effects of cannabinoids, where 10 mg/kg THC or CBD produces robust central effects without acute toxicity or profound motor impairment [23–26]. Due to the slow gastrointestinal absorption kinetics of oral gavage [27], EEG acquisition resumed 60 min after drug administration to ensure sufficient systemic availability. Throughout recordings, rats were allowed to move freely in their home cages, tethered to the amplifier *via* a lightweight cable. EEG signals were collected using the BrainScope system (M&I, Prague, Czech Republic) at 16-bit resolution (7.63 nV/bit; ~130 bit/μV), ±500 μV input range, and 1 kHz sampling rate. Behavior was continuously monitored by a trained observer who annotated behavioral states (active vs. inactive) online using predefined keyboard markers. Observers were blinded to treatment. If rats showed signs suggestive of sleep (immobility, eye closure), gentle handling was applied briefly to maintain wakefulness.

### EEG signal preprocessing

Preprocessing was performed in BrainVision Analyzer 2.1 (Brain Products GmbH). The EEG was re-referenced to the common average of twelve implanted electrodes (C3, C4, F3, F4, P3, P4, P5, P6, T3, T4, T5, T6). Signals were filtered using zero-phase Butterworth IIR filters: a 0.5 Hz high-pass filter (8th order; time constant 0.318 s), a 100 Hz low-pass filter (8th order), and a 50 Hz notch filter. All EEG data were visually inspected by an experienced analyst to remove gross artifacts, including malfunctioning electrodes and contaminated segments. Continuous data were then segmented into the predefined post-administration intervals (80–90 min, 110–120 min, 140–150 min). Within each interval, data were further segmented according to behavioral state (active vs. inactive). Each segment was subsequently divided into consecutive 2-s epochs while rejecting epochs containing artifacts. Only datasets containing at least 60 seconds of clean EEG in each experimental condition were included in further analysis. For source and connectivity analyses, the preprocessed EEG was exported in ASCII format. For all subsequent spectral, source, and connectivity analyses, only epochs corresponding to behaviorally inactive periods were retained and analyzed.

### Spectral power analysis

Spectral analysis was performed on 2-s epochs with 75 % overlap. Fast Fourier Transform (FFT) parameters were set to 0.5 Hz resolution, voltage (non-complex) output, and half-spectrum computation. A periodic, variance-corrected Hanning window (10 % of epoch length) was applied, and all segments were zero-padded before transformation. FFT was computed for every epoch and electrode, and power spectra were averaged across epochs for each condition and subject. Sensor-level spectra were plotted to illustrate the temporal evolution of treatment effects; however, these representations primarily served a descriptive purpose. Statistical inference was conducted exclusively in source space, where anatomical specificity enables meaningful comparison across treatment conditions. Statistical evaluation of spectral power differences across treatments was conducted using cluster-based permutation testing [28]. To identify neural generators of power modulations, source localization was performed using an FEM-based forward model (FieldTrip [29]) built on a homogeneous isotropic rat brain volume conductor with co-registered 12-electrode positions [30]. The brain volume was discretized at an isotropic resolution of 1 mm. Band-pass–filtered EEG (δ: 1–4 Hz, θ: 4–8 Hz, α: 8–12 Hz, β: 12–30 Hz, γ: 30–40 Hz) was segmented into 2-s epochs and projected onto a grid of dipoles using eLORETA [31]. For each voxel, a representative source-space time series was obtained as the first principal component of the three orthogonal dipole moments, and voxel-wise power was averaged across epochs.

### Global functional connectivity (GFC) analysis

Global functional connectivity (GFC) was computed for each frequency band from the source-projected EEG. The source time series were downsampled to 250 Hz for analysis. Connectivity was estimated using the orthogonalized amplitude envelope correlation (oAEC) method [32]. For each dipole pair, the tested dipole’s signal was first orthogonalized relative to the seed dipole to minimize volume conduction artifacts. Analytic amplitude envelopes were extracted via the Hilbert transform, and connectivity was defined as the absolute Spearman correlation between envelopes. GFC quantifies nondirected coupling strength, defined as the median orthogonalized amplitude envelope correlation between each seed voxel and a representative subset of all other dipoles, thereby reflecting the magnitude of global connectivity. To reduce the computational load, the set of tested dipoles for each seed was randomly downsampled eight times. GFC for each seed dipole was defined as the median connectivity across tested dipoles, yielding one GFC map per condition and frequency band.

### Statistical evaluation

Changes from baseline were evaluated using paired-sample t-tests comparing each post-administration interval against the baseline measurement. To obtain empirical p-values and ensure strict Type I error control, a non-parametric permutation test was applied: treatment labels (“baseline” vs. “time T”) were randomly permuted, and for each permutation the t-statistic was recalculated across 8000 iterations, forming a null distribution. The empirical p-value was defined as the proportion of permuted t-statistics exceeding the observed value. Multiple comparisons were corrected using the Benjamini–Hochberg false discovery rate (FDR), and corrected q-values (reported as p-values for simplicity) were mapped onto the TOHOKU Rat Brain Atlas™ [33] to identify anatomical regions exhibiting significant EEG power alterations.

## Results

### Spectral power analysis

**Fig. 1:**
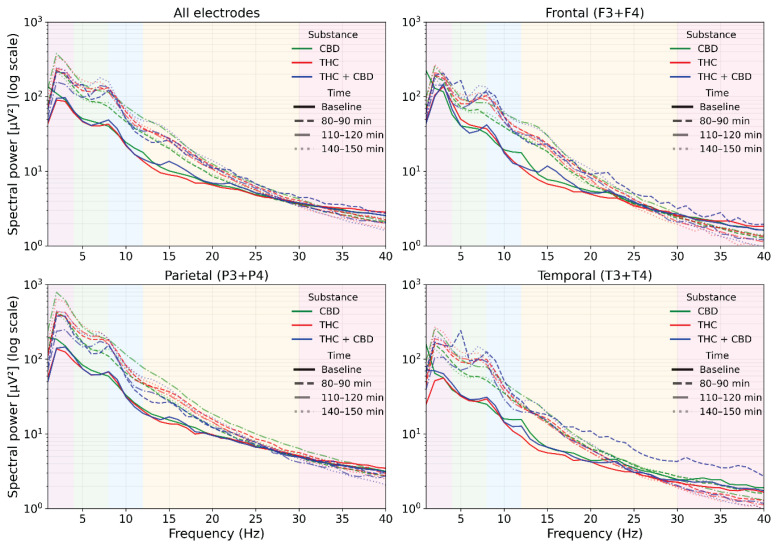
Spectral power (1–40 Hz) across cortical regions for each treatment and time point. Spectral power (μV^2^, log scale) averaged across electrode groups (All electrodes, Frontal (F3+F4), Parietal (P3+P4), Temporal (T3+T4)) is shown for three pharmacological conditions and four post-administration intervals. Each substance is plotted using a distinct color: CBD (green), THC (red), and THC + CBD (blue). Within each substance, time points are differentiated by line style: Baseline (solid line), 80–90 min (dashed line), 110–120 min (dash–dot line), and 140–150 min (dotted line). Vertical shaded bands indicate canonical frequency ranges (δ, θ, α, β, γ). Spectra are displayed on a logarithmic scale with shared axes.

Across conditions, spectra preserved the typical 1/f profile at the sensor level but were systematically shifted toward higher low-frequency power after drug administration. As shown in Figure 1, these spectral changes are presented purely descriptively to illustrate the overall temporal evolution of treatment effects across the three post-administration intervals. CBD produced modest, spatially consistent increases in δ–θ and low α bands, most evident at 80–90 min and stabilizing by 110–120 min, with minimal change above ~25 Hz. THC produced stronger low-frequency facilitation extending into the α/β range, accompanied by progressive attenuation in the upper band, resulting in the first clear reduction in power at ~30–40 Hz by 110–120 min, particularly over temporal and parietal leads. A partial return toward baseline was observed at 140–150 min. The THC+CBD combination resulted in the largest broadband δ–β enhancement with a marked high-β/low-γ depression, again most pronounced temporally and parietally. Based on these descriptive observations, the 110–120 min interval was selected a priori for source-level statistical analysis because it exhibited the most consistent and robust drug-related deviations across treatments and animals.

### Source-level spectral power analysis

CBD (10 mg/kg) predominantly increased low-frequency power across limbic–thalamo–auditory circuits, with only sparse decreases in high-frequency power. The strongest beta effects localized to the entorhinal–hippocampal loop and anterior thalamus (medial entorhinal: t = 4.53, p < 0.05; parasubiculum: t = 4.40, p < 0.05; postrhinal: t = 4.36, p < 0.05; anterodorsal thalamus: t = 4.21, p < 0.05; ventrolateral thalamus: t = 4.18, p < 0.05; MGB ventral/dorsal: t ≈ 4.2/4.2, p < 0.05).

**Fig. 2:**
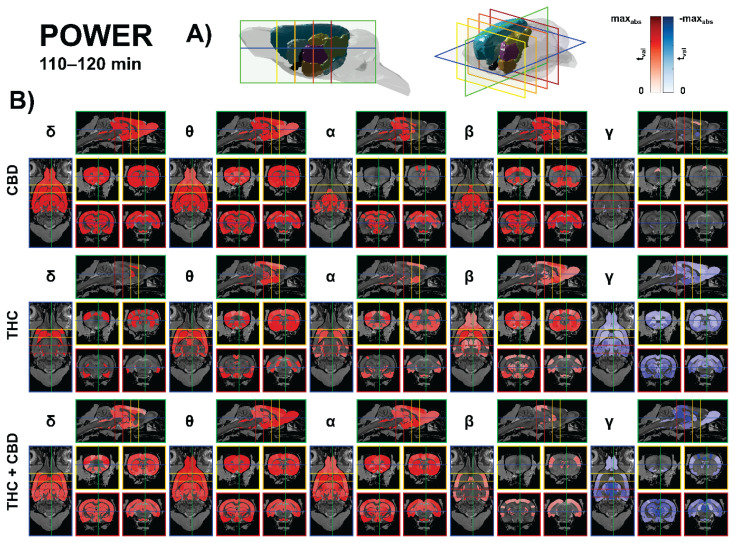
Source-level spectral power (110–120 min). **A)** 3D anatomical model of the rat brain indicating the displayed cutting planes: horizontal slice at −6 mm ventro-dorsally (blue), sagittal slice at +0.5 mm laterally (green), and four coronal slices at +3.5 mm (yellow), 0 mm (light orange), −3.5 mm (dark orange), and −7 mm (red) from bregma. Colored brain structures follow the TOHOKU Rat Brain Atlas™: Isocortex (light blue), Pallidum (black), Hippocampus (gold), Hypothalamus (coral), Diencephalon (purple), Midbrain (light green), Striatum (cyan). **B)** eLORETA maps of band-limited power change vs. baseline for CBD (10 mg/kg), THC (10 mg/kg), and THC+CBD (10+10 mg/kg) across δ (1–4 Hz), θ (4–8 Hz), α (8–12 Hz), β (12–30 Hz), and γ (30–40 Hz). Warm colors indicate increases; cool colors indicate decreases. Only FDR-corrected permutation-significant areas (q<0.05) are shown.

Theta rose in cingulate and hippocampal fields and reuniens/parataenial thalamus (Cg1: t = 4.21, p < 0.05; dentate gyrus: t = 4.10, p < 0.05; CA1: t = 4.03, p < 0.05). Along the auditory pathway, coherent theta/beta increases spanned cochlear nuclei → lateral lemniscus → inferior colliculus (e.g., IC external β: t = 4.27, p < 0.05; VCN anterior β: t = 4.26, p < 0.05). Gamma decreases were modest and brainstem-limited (e.g., SNr γ: t = −3.11, p < 0.05). Overall, CBD chiefly amplifies delta–theta–beta power within limbic–thalamic and auditory networks. The results are presented in Figure 2.

THC (10 mg/kg) reproduced low-frequency facilitation but introduced a large-scale gamma suppression centered on thalamo-mesencephalic and basal ganglia hubs. Alpha increased in auditory–vestibular brainstem and thalamus (nucleus sagulum: t = 4.39, p < 0.01; DCN deep core: t = 4.25, p < 0.01; vestibular apparatus: t = 4.14, p < 0.01; posterior intralaminar thalamus: t = 3.54, p < 0.01), while beta rose across striato-insular and thalamo-auditory nodes (claustrum: t = 4.37, p < 0.01; ventral striatum: t = 3.98, p < 0.01; caudate–putamen: t = 3.56, p < 0.01; MGB marginal: t = 3.63, p < 0.01). Theta also increased in fronto-striatal/limbic territory and early auditory relays (FA3: t = 3.90, p < 0.01; CPu: t = 3.30, p < 0.01). Dominant gamma suppression was widespread, peaking in LD thalamus (DM) (t = −9.16, p < 0.01), subthalamic nucleus (t = −8.23, p < 0.01), zona incerta (t ≈ −7.0 to −5.9, p < 0.01), substantia nigra/VTA (t ≈ −6.7 to −6.1, p < 0.01), hippocampus (CA2/CA3/CA1 t ≈ −6.1 to −4.0, p < 0.01), and along the entire auditory axis (lemniscal nuclei and IC; t ≈ −6.4 to −2.8, p < 0.01). The results are presented in Figure 2.

The THC + CBD (10 + 10 mg/kg) combination produced the broadest modulation, characterized by robust low-frequency gains and the most pervasive gamma suppression across the thalamus, midbrain relays, and somatosensory cortex. Theta increases encompassed secondary motor and frontal association cortex and subcortical reward/thalamic nodes (SMA: t = 4.63, p < 0.01; FA3: t = 4.21, p < 0.01; subthalamic nucleus: t = 4.23, p < 0.01; VTA: t = 3.95, p < 0.01; accumbens core/shell: t ≈ 3.96–3.97, p < 0.01) and extended along lateral lemniscus and inferior colliculus (t ≈ 3.8–3.1, p < 0.01). Alpha rose in vestibular apparatus and multiple thalamic/limbic parcels (e.g., posterior intralaminar: t = 4.12, p < 0.01; lateral entorhinal: t = 4.14, p < 0.01; CPu: t = 3.70, p < 0.05), while beta gains were smaller and centered on auditory transfer points (e.g., DCN deep core: t = 3.92, p < 0.01). Gamma power was strongly and widely reduced – S1 hindlimb (t = −5.74, p < 0.01), intralaminar/associative thalamus (AV-DM, CL, Pc, MD/VM/CM/PVT t ≈ −5.7 to −5.1, p < 0.01), lateral posterior (MR) (t = −5.53, p < 0.01), PAG and superior colliculus (t ≈ −5.3 to −5.1, p < 0.01), with additional declines in hippocampus (CA1/CA3: t = −4.02/−3.90, p < 0.01) and along the auditory pathway including primary auditory cortex (typical t ≈ −3.5 to −2.8, p < 0.01). Delta increases accompanied this profile in the thalamus/insula/olfacto-auditory nodes (representative thalamic parcels t ≈ 2.5–3.1, p < 0.01). The results are presented in Figure 2.

### Source-level global functional connectivity

**Fig. 3:**
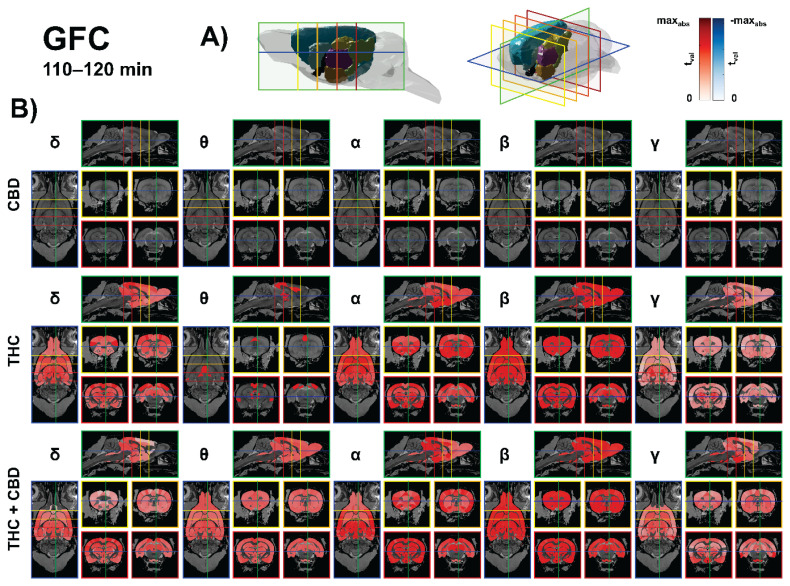
Source-level global functional connectivity (110–120 min). **A)** 3D anatomical model of the rat brain indicating the displayed cutting planes: horizontal slice at −6 mm ventro-dorsally (blue), sagittal slice at +0.5 mm laterally (green), and four coronal slices at +3.5 mm (yellow), 0 mm (light orange), −3.5 mm (dark orange), and −7 mm (red) from bregma. Colored brain structures follow the TOHOKU Rat Brain Atlas™: Isocortex (light blue), Pallidum (black), Hippocampus (gold), Hypothalamus (coral), Diencephalon (purple), Midbrain (light green), Striatum (cyan). **B)** Source-space GFC change vs. baseline for CBD (10 mg/kg), THC (10 mg/kg), and THC+CBD (10+10 mg/kg) across δ (1–4 Hz), θ (4–8 Hz), α (8–12 Hz), β (12–30 Hz), and γ (30–40 Hz). Warm colors indicate increases; cool colors indicate decreases. Only FDR-corrected permutation-significant areas (q<0.05) are displayed.

CBD (10 mg/kg) produced no source-level GFC clusters surviving FDR correction. In contrast, THC (10 mg/kg) selectively increased GFC, most prominently in gamma, centered on thalamic and basal ganglia relays with extensions to pretectal and auditory pathways—e.g., laterodorsal thalamus (DM) (t = 7.43, p < 0.01), anterodorsal thalamus (t = 6.84, p < 0.01), subthalamic nucleus (t = 6.82, p < 0.01), intramedullary lamina (t = 6.21, p < 0.01), lateral posterior thalamus (lateral) (t = 6.13, p < 0.01), posterior thalamus (t = 5.95, p < 0.01), pretectal region (t = 5.87, p < 0.01), VPL (t = 5.80, p < 0.01). Alpha rises predominated in olfactory–limbic–insular and striatal nodes (piriform L3: t = 5.80, p < 0.01; dysgranular insula: t = 5.33, p < 0.01; VPM: t = 5.27, p < 0.01; BNST: t = 5.22, p < 0.01). Delta effects were smaller (FA3: t = 4.94, p < 0.01; S1 hindlimb: t = 4.43, p < 0.01), and theta increases were modest and scattered across midline thalamus and retrosplenial cortex (fasciola cinereum: t = 3.48, p < 0.05 IMD: t = 3.26, p < 0.05; PVT: t = 3.25, p < 0.05; V1: t = 3.22, p < 0.05). The results are presented in Figure 3.

The THC + CBD combination (10 + 10 mg/kg) produced the broadest GFC augmentation, with strongest delta increases peaking in posterior–parietal and early sensory hubs and extending to hippocampo-thalamic and collicular relays (Parietal association, posterior: t = 9.72, p < 0.01; S1 barrel: t = 8.00, p < 0.01; V2 medial: t = 7.94, p < 0.01; CA1: t = 7.28, p < 0.01; LP thalamus (lateral): t = 7.13, p < 0.01). Beta increases centered on associative/sensory thalamus, basal ganglia, and auditory nodes (VA: t = 6.76, p < 0.01; VPLpc: t = 6.71, p < 0.01; SN lateral: t = 6.70, p < 0.01; VPM: t = 6.56, p < 0.01; MGB dorsal: t = 5.59, p < 0.01; A1: t = 5.98, p < 0.01). Alpha rose in insula/limbic–thalamic–auditory parcels (agranular insula, posterior: t = 6.06, p < 0.01; IC central: t = 5.74, p < 0.01; A1: t = 5.49, p < 0.01), theta engaged vestibular–auditory brainstem and midline thalamus (vestibular apparatus: t = 5.40, p < 0.01; DCN deep core: t = 5.28, p < 0.01; reuniens: t = 3.59, p < 0.05), and gamma showed smaller but reliable gains in associative cortex and brainstem relays (Parietal association, medial: t = 5.95, p < 0.01; pontine nuclei: t = 5.26, p < 0.01). Collectively, THC boosts GFC chiefly in gamma within thalamo-basal-auditory circuits, whereas THC + CBD amplifies GFC most in delta and beta with wide thalamic/auditory engagement and ancillary alpha/theta/gamma increases. The results are presented in Figure 3.

## Discussion

The present study demonstrates that the acute oral administration of THC (10 mg/kg), CBD (10 mg/kg), or their combination produces pronounced and long-lasting effects on EEG spectral power across the delta to gamma bands in rats. Source localization revealed that these effects were spatially specific, with the combination treatment displaying a largely THC-driven profile. A principal finding is the robust enhancement of lower-frequency spectral power (delta through beta), accompanied by a reduction in high-frequency (gamma) activity, indicating that cannabinoid exposure exerts bidirectional effects on EEG.

Human studies provide convergent support for cannabinoid-associated increases in lower-frequency power. Chronic cannabis users show elevated resting alpha (8–12 Hz), particularly in prefrontal regions, as revealed by EEG source imaging [10]. Observational studies in young adult cannabis users have similarly reported increased alpha and beta activity in prefrontal, frontal, and temporal areas during acute consumption [11]. In clinical populations, combined THC (10 mg) and CBD (200 mg) administered to insomnia patients increased regional alpha and beta activity during REM sleep [12]. These findings collectively indicate that cannabinoids can augment slower oscillations, particularly in prefrontal networks, under specific behavioral or physiological states. Rodent studies also demonstrate cannabinoid-induced enhancements in slower oscillations. Synthetic cannabinoid administration has been shown to prominently increase 5–6 Hz theta power [34]. Additionally, CB1 receptor agonists have been demonstrated to affect oscillatory activity in a region-dependent manner, reducing power in the 0.1–30 Hz range in hippocampal CA1 while altering power in the 30–100 Hz range in the medial prefrontal cortex in the same animals [35]. Our observation of widespread delta-to-beta increases after oral THC and CBD suggests that slow-frequency enhancement is a core electrophysiological consequence of acute cannabinoid action, not restricted to specific synthetic compounds, chronic exposure, or non-physiological preparations.

Mechanistically, these low-frequency increases likely arise from CB1 receptor-mediated shifts in excitatory–inhibitory balance. CB1 receptors are abundant in the prefrontal cortex, hippocampus, and other limbic structures [3], where their activation suppresses glutamatergic input to pyramidal cells and GABAergic input to fast-spiking interneurons [36]. This dual suppression can disrupt the precise temporal coordination required for high-frequency oscillations while simultaneously promoting slower, more synchronized network states. Indeed, slice physiology studies have demonstrated that CB1 receptor activation reduces firing precision and dampens gamma oscillations in hippocampal networks by suppressing excitatory drive [36]. We propose that the delta-to-beta power increase observed in our study reflects a shift in network dynamics toward slower, more synchronized oscillatory states, resulting from the CB1-mediated reduction of fast inhibitory and excitatory transmission.

Our source localization revealed that cannabinoid-induced power increases were most prominent in the prefrontal cortex, cingulate cortex, hippocampus, and striatal regions, including the nucleus accumbens. These findings are highly consistent with the known distribution of CB1 receptors in the rat brain, which are particularly dense in cortical regions, the hippocampus, the basal ganglia, and the cerebellum [37]. Prefrontal cortex emerged as a major locus of cannabinoid action, consistent with human studies showing THC-induced alterations in prefrontal dynamics and chronically elevated prefrontal alpha power in cannabis users [10,38]. Given the role of prefrontal oscillations in working memory, executive function, and cognitive control, these findings align with the cognitive impairments commonly observed after acute cannabinoid exposure [13].

Hippocampal involvement was also prominent. With among the highest CB1 densities in the brain [3], the hippocampus exhibits well-documented cannabinoid-induced disruptions, including reduced theta power, altered theta–gamma coupling, and impaired spatial memory [39]. Our localization of cannabinoid-induced changes to hippocampal structures is consistent with these findings and may relate to cannabis-induced impairments in memory processes, given the fundamental role of hippocampal theta in memory encoding and retrieval [40].

Striatal regions, including the nucleus accumbens, also showed substantial involvement. Previous rodent studies using multi-site local field potential recordings have demonstrated that acute THC suppresses gamma power in nucleus accumbens, dorsal striatum, and cingulate cortex, with these effects emerging approximately 2 hours post-administration and persisting up to 24 hours [17]. Similarly, vaporized THC has been shown to reduce gamma power and coherence in dorsal striatum-prefrontal circuits [16]. The present results extend these findings by demonstrating that oral cannabinoid administration produces frequency-specific, anatomically localized effects within striatal circuits, as determined using surface EEG source modelling. Given the importance of striatal networks in reward, motivation, and motor output, these alterations may contribute to cannabinoid-induced behavioral changes.

The cingulate cortex emerged as another prominent region in our source localization analyses. The cingulate is richly innervated by CB1 receptors and plays important roles in attention, emotion regulation, and cognitive control [41]. Previous studies have reported that edible THC produces time-dependent and sex-dependent gamma power suppression in rat cingulate cortex [17], and human neuroimaging work has shown that THC attenuates subgenual anterior cingulate cortex reactivity during negative affect induction [42]. Our localization of cannabinoid effects to the cingulate cortex provides a neurophysiological substrate for understanding how cannabinoids modulate emotional processing and attentional control.

Notably, our source localization also identified extensive engagement of auditory pathway structures, including cochlear nuclei, lateral lemniscus, inferior colliculus, and thalamo-auditory nodes. This anatomical finding provides a direct substrate for understanding THC-induced disruptions of auditory steady-state responses documented in human studies [15], suggesting that these sensory processing deficits reflect cumulative alterations across multiple stages of the auditory pathway rather than dysfunction at a single locus.

While our primary findings center on delta-to-beta power increases, we also observed an intriguing dissociation between gamma power suppression and enhanced gamma-band functional connectivity following THC administration. THC produced large-scale gamma power suppression in thalamo-mesencephalic and basal ganglia hubs, yet simultaneously increased gamma-band functional connectivity in these same regions. This dissociation likely reflects distinct mechanisms: local gamma power depends on the balance between excitation and inhibition provided by fast-spiking interneurons [43], while functional connectivity reflects phase synchronization between distant regions [44]. CB1-mediated reduction in excitatory drive may decrease local gamma amplitude while paradoxically increasing inter-regional phase coherence by constraining temporal dynamics across network nodes [45]. CBD alone produced minimal gamma changes and no significant alterations in functional connectivity, indicating that this dissociation is specifically mediated by CB1 receptor activation.

Importantly, our findings reveal that the regional pattern of cannabinoid effects was largely similar across THC, CBD, and their combination, though with differences in magnitude. The combination treatment produced a profile that was clearly THC-driven, with CBD exhibiting similar directionality but attenuated effects. This regional consistency across treatments suggests that, despite their distinct receptor pharmacology, THC and CBD may converge on common downstream network mechanisms. The similar regional localization may reflect shared influences on endocannabinoid signaling – THC through direct CB1 activation and CBD through inhibition of anandamide degradation and modulation of CB1-mediated signaling [2]. The dominance of THC in the combination profile likely reflects its more potent and direct action at CB1 receptors compared to CBD’s indirect and multi-target mechanisms.

Several limitations should be acknowledged. First, only male rats were used, which prevents assessment of potential sex-dependent variability in cannabinoid responses. Second, no dedicated behavioral tasks were included. However, by focusing exclusively on resting-state-like EEG, we examined a signal modality with high translational validity, directly comparable to human qEEG and resting-state analyses commonly used in clinical research. Third, both THC and CBD were tested at a single acute oral dose. While this precludes assessment of dose–response relationships, the use of equivalent doses follows prevailing experimental practice, thereby supporting cross-species comparability. The relatively subtle effects of CBD observed here may therefore reflect either dose-dependent sensitivity or the fact that CBD-related network changes are less readily captured under resting-state conditions. Finally, the acute design does not address potential adaptations that may arise from repeated exposure.

## Conclusions

Our study demonstrates that acute oral cannabinoid administration produces robust, regionally specific increases in delta-to-beta EEG spectral power in rats, with effects primarily localized to the prefrontal cortex, hippocampus, striatum, and cingulate cortex. The combination of THC and CBD produces a predominantly THC-driven profile, with CBD exhibiting similar but attenuated effects. These findings advance our understanding of cannabinoid neurophysiology by revealing the regional architecture underlying cannabinoid-induced oscillatory changes and by demonstrating that increases in lower frequency bands represent a core feature of cannabinoid action.
